# Predictive Prognostic Factors in Non-Calcific Supraspinatus Tendinopathy Treated with Focused Extracorporeal Shock Wave Therapy: An Artificial Neural Network Approach

**DOI:** 10.3390/life14060681

**Published:** 2024-05-25

**Authors:** Gabriele Santilli, Mario Vetrano, Massimiliano Mangone, Francesco Agostini, Andrea Bernetti, Daniele Coraci, Marco Paoloni, Alessandro de Sire, Teresa Paolucci, Eleonora Latini, Flavia Santoboni, Sveva Maria Nusca, Maria Chiara Vulpiani

**Affiliations:** 1 Physical Medicine and Rehabilitation Unit, Sant’Andrea Hospital, Sapienza University of Rome, 00189 Rome, Italy; 2 Department of Anatomical and Histological Sciences, Legal Medicine and Orthopedics, Sapienza University, 00185 Rome, Italy; 3 Department of Biological and Environmental Science and Technologies, University of Salento, 73100 Lecce, Italy; 4 Department of Neuroscience, Section of Rehabilitation, University of Padua, 35122 Padua, Italy; 5 Physical and Rehabilitative Medicine, Department of Medical and Surgical Sciences, University of Catanzaro “Magna Graecia”, 88100 Catanzaro, Italy; 6 Research Center on Musculoskeletal Health, MusculoSkeletalHealth@UMG, University of Catanzaro “Magna Graecia”, 88100 Catanzaro, Italy; 7 Department of Oral Medical Science and Biotechnology, G. D’Annunzio University of Chieti-Pescara, 66100 Chieti, Italy

**Keywords:** prognostic factors, supraspinatus, rotator cuff, shoulder, rehabilitation, ESWT, shockwaves, ultrasound, artificial neural network, machine learning

## Abstract

The supraspinatus tendon is one of the most involved tendons in the development of shoulder pain. Extracorporeal shockwave therapy (ESWT) has been recognized as a valid and safe treatment. Sometimes the symptoms cannot be relieved, or a relapse develops, affecting the patient’s quality of life. Therefore, a prediction protocol could be a powerful tool aiding our clinical decisions. An artificial neural network was run, in particular a multilayer perceptron model incorporating input information such as the VAS and Constant–Murley score, administered at T0 and at T1 after six months. It showed a model sensitivity of 80.7%, and the area under the ROC curve was 0.701, which demonstrates good discrimination. The aim of our study was to identify predictive factors for minimal clinically successful therapy (MCST), defined as a reduction of ≥40% in VAS score at T1 following ESWT for chronic non-calcific supraspinatus tendinopathy (SNCCT). From the male gender, we expect greater and more frequent clinical success. The more severe the patient’s initial condition, the greater the possibility that clinical success will decrease. The Constant and Murley score, Roles and Maudsley score, and VAS are not just evaluation tools to verify an improvement; they are also prognostic factors to be taken into consideration in the assessment of achieving clinical success. Due to the lower clinical improvement observed in older patients and those with worse clinical and functional scales, it would be preferable to also provide these patients with the possibility of combined treatments. The ANN predictive model is reasonable and accurate in studying the influence of prognostic factors and achieving clinical success in patients with chronic non-calcific tendinopathy of the supraspinatus treated with ESWT.

## 1. Introduction

The supraspinatus tendon is one of the most involved tendons in the development of shoulder pain, and its inflammation is a common condition causing joint dysfunction [1,2,3,4]. It is characterized by pain-related inflammation, leading to compromised performance and functionality [5,6,7]. The etiology of rotator cuff tendinopathy is multifactorial, involving both intrinsic and extrinsic mechanisms [8]. Diagnosis of supraspinatus pathology relies on patient history and physical examination, with commonly used and reliable signs including the drop arm test, empty can test, full can test, and shoulder lift-off sign [9]. Various imaging tools such as musculoskeletal ultrasound and magnetic resonance imaging (MRI) can aid in diagnosing rotator cuff (RC) disorders [10].

Several treatments are available depending on disease severity and the characteristic of the pathology as the presence of tendon tears, including rehabilitative training, physical therapy, non-steroidal anti-inflammatory drugs, and even local injective treatment or surgical treatment [11,12,13]. Nowadays, extracorporeal shockwave therapy (ESWT) has been recognized as a valid and safe treatment for supraspinatus tendinopathy and other rotator cuff-tearing pathologies [14]. The extensive use of ESWT can be attributed to several key factors: its clinical success rate [15], patient and physician satisfaction with minimal treatment sessions, short therapeutic sessions, cost-effectiveness, improved accessibility in recent years, and the absence of restrictive measures in daily activities during therapy.

Following treatment, symptoms of supraspinatus tendinopathy are generally alleviated, but sometimes the symptoms cannot be relieved, or a relapse develops, affecting the patient’s quality of life [16]. Therefore, a prediction protocol could be a powerful tool aiding clinical decisions. To develop the prediction protocol, we decided to use an artificial neural network (ANN), incorporating input information such as the Visual Analog Scale (VAS) and Constant–Murley score (CMS), often used to assess patients in terms of shoulder pain and function and to evaluate treatment effects [17,18], along with a modified Roles and Maudsley score (RM) [19]. However, current research has indicated that VAS and CMS, along with traditional statistics, need assistance to accurately assess treatment effects and prognosis [20,21]. VAS and CMS are subjective and have limitations such as individual variability, psychological influences, and biases towards social desirability.

The predictive model utilizing the ANN is both reasonable and accurate in examining the impact on minimal clinically successful therapy in tendinopathy treated with ESWT [22]. In the field of physical medicine and rehabilitation, various machine-learning approaches have been developed in recent years across several subfields of the discipline [23,24,25,26]. Therefore, evaluating an ANN may be a solution to ensure a better and more accurate prognosis, integrating medical and physical examination and also including RM score assessment to evaluate patient pain compared to usual daily life activities [27]. The ANN correlates patient data, including demographic information such as age, sex, and affected side, with factors such as tendon degeneration, gender differences, and dominant side all related to overuse, a significant risk factor for tendinopathy. Utilizing the ANN offers numerous advantages: (1) improved clinical decisions and personalized therapy, providing patients with precise prognosis information and treatment success probabilities; (2) reduction in healthcare costs by effectively identifying therapy candidates, avoiding unnecessary treatments; (3) accelerated clinical research to identify ideal therapeutic candidates; and (4) enhanced healthcare equity, ensuring tailored care for diverse needs, reducing disparities and ultimately positively impacting public health and healthcare system efficiency. The aim of our study is to identify predictive factors for minimal clinically successful therapy (MCST) following ESWT for chronic non-calcific supraspinatus tendinopathy (SNCCT).

## 2. Materials and Methods

### 2.1. Study Design and Population

**Figure 1:**
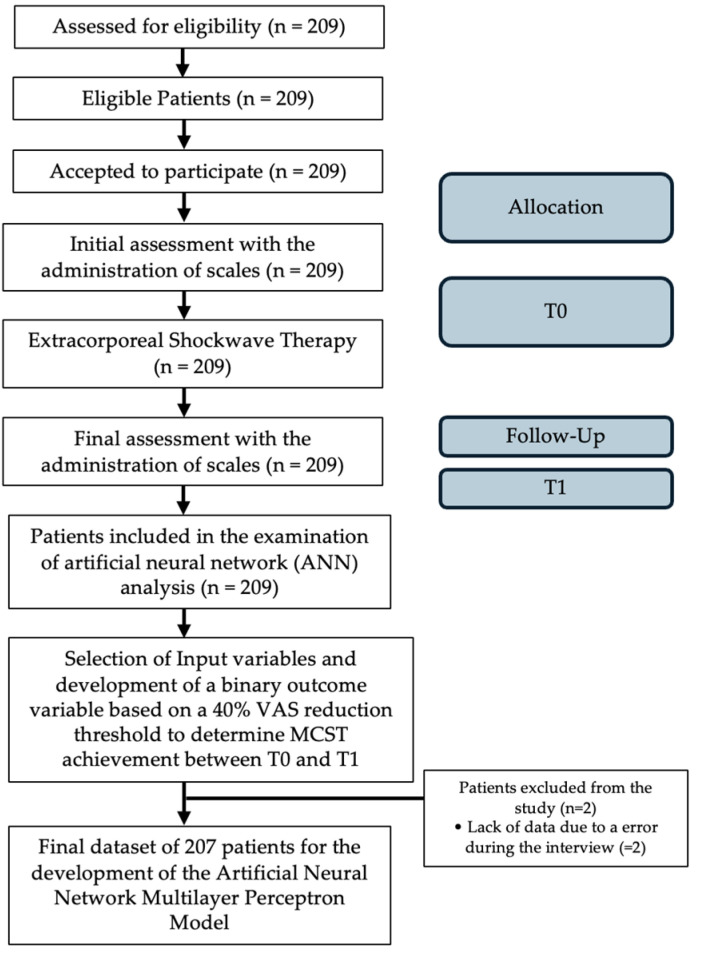
Flow diagram of the study.

This observational study follows good clinical practice and the ethics of the Helsinki Declaration, approved by La Sapienza University’s Institutional Review Board (RS 6532/2021—Approval Date: 15 October 2021). Informed consent forms were signed by all patients, and the data have been anonymized. Data of patients treated in our institution ”AOU Sant’Andrea of Rome” for symptomatic SNCCT between 2011 and 2020 were retrospectively collected and analyzed. Supraspinatus non-calcific chronic tendinopathy (SNCCT) was diagnosed based on clinical symptoms, physical examinations, and imaging studies. All the patients who fulfilled the following selection criteria were considered eligible: (1) between 25 and 85 years old; (2) painful lateral aspect of shoulder and pain exacerbation with overhead activity; (3) having local pain in the area of the shoulder; (4) symptoms present from the last three months; (5) both genders; (6) reduced range of motion (ROM); (7) positive tendinopathy rotator cuff test. Patients were excluded if they had any of the following: (1) marked atrophy or weakness of any shoulder girdle muscle, (2) previous surgery, (4) recent corticosteroid use or nerve blockage, (5) tumor in the treatment area, (6) pregnancy, or (7) coagulation abnormalities. All eligible patients completed a demographic and clinical questionnaire that assessed age, gender, affected side, and duration of symptoms, and the scales administered at T0 and at T1 after six months from ESWT were the RM score, VAS score, and CMS. A flow diagram of the study is shown in Figure 1.

### 2.2. Intervention

The study protocol used was in line with the current state of the art in treating SP with ESWT performed by the principal authors with the Modulith SLK system (Storz Medical, Tagerwilen, Switzerland), with an electromagnetic extracorporeal shockwave generator equipped with an in-line ultrasound positioning system on the target zone at 2 cm from the insertion point of the supraspinatus tendon on the greater tuberosity. All treatments were performed with no local anesthesia. Participants underwent ESWT by lying on a bed with the affected arm positioned in adduction, the elbow flexed at 90 degrees, and the hand under the homolateral gluteus. All participants received ESWT at an energy level of 0.20 mJ/mm^2^, 2400 pulses, and 6 Hz of frequency once a week for three weeks [21]. The VAS, CMS, and RM were administered before treatment and at 6 months after the end of the ESWT. All patients completed a 24-week follow-up (T1). To identify patients who benefited from ESWT, the therapeutic success or MCST was defined as a reduction of ≥40% in VAS score at T1 compared to the baseline. This percentage value of reduction in VAS was chosen as MCST because it represents a value observed in numerous clinical studies concerning supraspinatus tendinopathy treated with ESWT [28,29,30,31].

#### Outcomes

The Visual Analog Scale (VAS) comprises a 100 mm horizontal line, with “no pain” denoted at the left end (score: 0) and “pain as severe as possible” at the right end (score: 10). Patients were instructed to place a hatch mark on the line corresponding to their current pain level, both at rest and during their most painful movement. The VAS score was subsequently determined by measuring the distance in millimeters between the left endpoint and the patient’s mark [17].

To assess symptom severity and patient functionality, the CMS score questionnaire translated and validated for Italian was applied [32]. This is a 100-item instrument used to provide a clinical assessment of the shoulder in terms of pain severity (maximum score of 15), the ability of the patient to perform activities of daily living (maximum score of 20), active ROM (maximum score of 30), and shoulder strength (maximum score of 25). On this scale, a score of 0 indicates that the patient has intense pain and is unable to perform activities of daily living using the shoulder in question. The maximum score of 100 indicates that the patient is pain-free and able to execute all the activities of daily living. This instrument is a compound score containing four subscales: two self-reported subscales and two scales administered by an external rater. Normal values are between 70 and 90 points [33].

The RM score was used to evaluate the patient’s pain in relation to normal daily activities. An RM score of 1 represented excellent quality of life (i.e., no symptoms; unlimited walking ability without pain; patient satisfied with the treatment outcome [when assessed after RSWT or placebo]), an RM score of 2 represented good quality of life (i.e., ability to walk more than one hour without pain; symptoms substantially decreased after treatment; patient satisfied with the treatment outcome), an RM score of 3 indicated acceptable quality of life (i.e., inability to walk more than one hour without pain; symptoms somewhat better and pain more tolerable than before treatment; patient slightly satisfied with the treatment outcome), and an RM score of 4 indicated poor quality of life (i.e., inability to walk without severe pain; symptoms not better or even worse after treatment; patient not satisfied with the treatment outcome) [19].

### 2.3. Artificial Neural Networks

The ANN model was developed using SPSS 27.0 statistical software by SPSS Inc. in Chicago, IL, USA [34]. The ANN analysis aimed to identify influential variables and model their impact on MCST over a 6-month follow-up period. The chosen model was the multilayer perceptron (MLP), which comprises three layers: the input layer, hidden layer, and output layer. The MLP ANN utilized predictive factors within the input layer (age, gender, affected side, RM score, VAS, CMS) and the output layer (attainment of MCST or not) to learn the intricate relationship between inputs and outputs. The output layer featured two neurons with a target error of 0.0001, a learning rate of 0.001, and a maximum training period of 1000 iterations, and training concluded upon reaching the minimum error value. In our study, patients were randomly assigned to two groups: 70% for the total training sample and 30% for the validation sample (testing group) [35]. These subsets were employed in developing the ANN models. After training the MLP ANN, it was employed to predict outcomes using the test subset.

### 2.4. Statistical Analysis

Power analysis was performed (G*Power, v.3.1.9.2, Franz Faul, Germany). Based on the study of Zhang YF et al. [36], a desired statistical power of 80% was assumed to detect a 2-point difference with a standard deviation (SD) of 1.5 points in the VAS pain score based on a 2-tailed t-test with a Bonferroni significance level of α  =  0.05. A drop-out rate of 10% was allowed in these conditions. With these parameters, 19 participants were needed. Differences in VAS, CMS, and RM scores between baseline and the follow-up were assessed using a paired samples t-test. We calculated the mean and standard deviation (SD) of interval data, the frequency of categorical data, and the bivariate correlations, and *p* < 0.05 was considered statistically different. Receiver operator characteristic (ROC) curve analysis was created and used to calculate the specificities, positive predictive value, and negative predictive value of the ANN. Furthermore, to assess the performance of the ANN, we used various evaluation metrics: the overall accuracy, the precision, the recall, and the F1-Score [37].

## 3. Results

### 3.1. Patient Demographic and Clinical Characteristics

All patients had symptoms for at least three months. To identify who would benefit from ESWT therapy, we inserted a minimum clinically successful therapy (MCST) that was defined as a reduction of ≥40% in VAS score at T1 compared to the baseline [28,29,30,31]. One hundred thirty-eight patients (66% of the total) who reached the threshold value were identified and denoted with a “1” and were included together with the remaining patients who did not reach this clinical improvement, patients identified with “0” in a binary binomial value, as the dependent variable for the ANN. In Table 1, we present the demographic and clinical characteristics of the patients, divided into two groups after applying a threshold of a 40% decrease in VAS after ESWT therapy at 6 months. Group 1 corresponds to those who have reached the MCST, and group 0 to those who have not reached the MCST. An independent samples t-test was conducted to compare continuous variables between the two groups. The results are as follows. The mean age of group 1 was 58.9 years (SD = 10.1), compared to a mean age of 60.6 years (SD = 10.5) for group 0. The difference was statistically not significant, *p* = 0.265. The mean score for VAS in group 1 was 6.1 (SD = 1.6), whereas it was 6.1 (SD = 1.9) for group 0. This difference was statistically not significant at *p* = 0.96. The mean score for CMS in group 1 was 64.6 (SD = 15.1), whereas it was 62.1 (SD = 16.7) for group 0. This difference was statistically not significant at *p* = 0.28. For categorical variables, a Chi-square test of independence was performed. The results are as follows. There were 61 males and 77 females in group 1 and 27 males and 44 females in group 0. The difference was statistically not significant, χ^2^ = 0.73, *p* = 0.392. The affected side was the left side for 56 participants and the right side for 82 participants in group 1, compared to 25 on the left and 46 on the right in group 0. The difference was statistically not significant, χ^2^ = 0.56, *p* = 0.45. In all four grades of Roles and Maudsley, there were no statistically significant differences between the groups, *p* = 0.3. Given the homogeneity of the two groups, the results imply that the factors determining the success of the therapy might go beyond linear relationships between basic demographic and clinical characteristics. They could potentially involve more complex interactions or less obvious variables, which an artificial neural network can capture, allowing for the personalization of treatment plans and optimizing resources in healthcare. This approach can lead to better patient outcomes and more efficient clinical practices. No significant adverse effect related to ESWT was found. We decided to include the following inputs in Table 2 in the ANN because they are the ones we can acknowledge at the beginning before undertaking therapy to develop a future prognostic prediction. Table 3 shows the sample distribution of the ANN model; from the dataset of 209 patients, two patients were excluded from the algorithm calculation due to missing values in the Roles and Maudsley scale resulting from an error during the data collection procedure. With traditional statistical analysis, we observed the following: a total of 209 eligible patients with supraspinatus non-calcific chronic tendinopathy (SNCCT) at our institution “AOU Sant’Andrea of Rome” between 2011 and 2020 were included in the study, 88 males and 121 females, with an average age of 59.5 years, with a ± 10.2 SD and 128 at the right shoulder and 81 at the left shoulder. The baseline average for VAS, CMS, and RM were, respectively, 6.1 points with ±1.7 SD, 63.8 points with ±15.6 points SD, and 2.2 points with ±0.9 SD (Table 4). At the last follow-up at T1, the VAS, CMS, and RM had an average, respectively, of 3.1 points with ±2.3 SD, 79.8 points with ±16 points SD, and 1.2 points with ±0.7 SD. We detected a positive Pearson bivariate correlation between the VAS at T0 and the VAS at T1 (r = 0.42, *p* = 0.001), a positive Pearson bivariate correlation between the CMS at T0 and the CMS at T1 (r = 0.47, *p* = 0.001), and a bivariate correlation of Spearman positive between RM at T0 and RM at T1 (r = 0.38, *p* = 0.001).

### 3.2. ANN Model Analysis

The model developed the interrelationships between predictor variables (input nodes), hidden variables (seven items in one hidden layer), and MCST. The six predictive factors are summarized in Table 5. Bivariate correlations of all factors were performed between these and the outcome MCST [38,39]. Pearson correlation was performed for parametric values, and Spearman Rho correlation for non-parametric values. The importance, normalized importance, and value of the six independent variables are illustrated in Table 6. Specifically, a lower age increased the odds of MCST, a higher CMS increased the odds of MCST (*p* < 0.01), a lower VAS increased the odds of MCST (*p* < 0.05), a lower RM increased the odds of MCST (*p* < 0.05), and male gender increased the odds of MCST (*p* < 0.01).

### 3.3. Accuracy of the Model

The developed ANN model has a sensitivity of 80.7%, a specificity of 72.3%, and a negative predictive value of 96.3%. The area under the ROC curve was 0.701, which demonstrated good discrimination. Table 7 shows the value of the area under the ROC curve and the predictive values of ANN models to predict the achievement of MCST. Furthermore, to assess the performance and accuracy of the ANN, we used evaluation metrics: the overall accuracy was 74.6%, precision was 62.7%, the recall was 95.4%, and the F1-Score was 75.6% [37].

## 4. Discussion

We have chosen to use the ANN over other algorithms because it is particularly well suited for capturing complex and nonlinear relationships in the data [40]. In our case, the relationships between the inputs and clinical success are intricate and challenging to model with simpler algorithms such as decision trees or support vector machines. In addition, the ANN is capable of automatically learning which relationships are most significant, and in our medical field, this is crucial for guiding prognosis.

In physical and rehabilitation medicine, we have numerous and diverse instrumental physical therapies, also mediated and not by physiotherapists. In the field of rehabilitation, it is not always possible to provide a probable prognosis to patients, but we rely on empirical experience; guidelines indicate the path to follow without giving a probabilistic percentage of success, which is often of greater interest to the patients. The knowledge of the prognostic factors leads physiatrists, orthopedic surgeons, and physiotherapists to better understand the extent of recovery, consequently adapting the rehabilitation path and the patient’s expectations [41]. Thanks to these tools, we may be more precise in prognosis in the future. There are limited studies providing evidence on specific patient factors that may lead to better outcomes with ESWT. One observational study evaluated characteristics and outcomes for patients treated with ESWT for various tendinopathies. In general, they found no significant correlation between blood type, occupation, specific sports activity, location of the tendinopathy, or the presence of comorbidities, but it was found that the male gender may be a positive prognostic factor for ESWT across multiple tendon pathologies, including rotator cuff tendinopathy, Achilles tendinopathy, patellar tendinopathy, or plantar fasciitis [42]. In another study about ESWT for RC tendinopathy, the authors found that males tended to do better than females with regard to improved pain and function [43]. In our findings, we can confirm those results, thanks to a significant strong correlation (*p* < 0.001) between the male gender and a significative decrease in the VAS score. In a recent Cochrane review, it was noted that the effect of ESWT on RC tendinopathy remains uncertain [44], but other studies, such as an RCT [45] that evaluated the effectiveness of ESWT compared to sham ESWT, demonstrated the effectiveness of this therapy; in previous studies, it was hypothesized that the beneficial effect of ESWT existed up to a 12-month follow-up [28,43,46]. Our results allow us to state that ESWT is a safe and effective therapy for SNCCT in the long term; in fact, significant improvements were observed in the six months following therapy, as shown in Table 4: a reduction in VAS score, from a mean of 6.1 ± 1.7 to 3.1 ± 2.3 (*p* = 0.000); an increase in CMS score, with the mean rising from 63.8 ± 15.6 to 79.8 ± 16 (*p* = 0.000); and a decrease in the RM score, with the mean changing from 2.2 ± 0.9 to 1.2 ± 0.7 (*p* = 0.000). We also found that the higher the initial VAS, the higher it will be at six months, and therefore, the patient who has more pain will have less margin for improvement compared to the one who already had less pain initially. In a similar way, this applies to CMS and RM at a functional level. We investigated the prognostic factors influencing minimum clinically successful therapy (MCST) at six months after ESWT for patients with supraspinatus non-calcific chronic tendinopathy (SNCCT). The ANN model was useful for guiding authors in recommending ESWT, with high predictive ability and an anticipation of the outcome based on patients’ characteristics [22]. The results revealed that age before ESWT was the most influential factor in deciding the probability of success of MCST; this finding agrees with another paper, cited in a recent review [47], where it was noted that the greater probability of success had been achieved by the youngest participant [48]. However, the CMS score, VAS, and RM at the time of the ESWT were also very influential factors in deciding the probability of success of the minimum clinically successful therapy (MCST), that is, a reduction in VAS of 40% from the baseline. Considering our results, the values of the CMS, RM, and VAS clinical scales should be considered decisive in identifying a possible minimum clinically successful therapy (MCST) with good probability through the artificial neural network.

Using the ANN, we observed in our sample a more effective response to ESWT when the main predictive factors, expressed in clinical and functional scales, were better and the patients were male and younger. Based on our findings, we suggest considering a combined therapy [29,49,50,51,52,53,54,55,56,57,58] for patients who are older, female, or those presenting with worse scores on clinical and functional scales at the time of outpatient assessment.

Among the numerous biological effects of ESWT [59,60,61,62,63,64,65], several morphological changes manifest as evident clinical improvements in most tendinopathies treated with ESWT. Therefore, a clear improvement in clinical scales is justified. Thanks to AI, though, and in this paper, specifically to the ANN, we should now also consider that clinical scales, such as the Constant and Murley, the Roles and Maudsley, and the VAS, are not just an evaluation tool to verify an improvement, but these clinical scales could be also considered as prognostic factors to be taken into consideration in the assessment of achieving clinical success.

With regard to limitations and biases, firstly, the sample size of 209, though adequate for evaluating variables for the ANN model, should be expanded for DM studies to ensure effectiveness. Secondly, not all potentially significant variables were identified to predict ESWT efficacy. Future studies should include additional factors such as interval, impulses per second, BMI, and comorbidities like diabetes. Lastly, longer-term follow-up, up to 12 or 24 months, should be considered, possibly via telephone, due to patient difficulty in returning.

## 5. Conclusions

Taken together, the results of our study showed that data mining could be considered an effective tool in predicting the clinical success of a therapy. In physical and rehabilitation medicine, where therapies are recent, and there is a lack of clear evidence on prognosis, machine learning will help us to make more precise predictions for patients. Thanks to machine learning, patients will be able to make choices that are more in line with the therapies proposed by doctors. From the male gender, we expect greater and more frequent clinical success with ESWT for chronic non-calcific tendinopathy of the supraspinatus. The more severe the patient’s initial condition, the greater the possibility that clinical success will decrease. ESWT is a safe and effective therapy for chronic non-calcific tendinopathy of the supraspinatus in the long term; the Constant and Murley score, Roles and Maudsley score, and the VAS are not just evaluation tools to verify an improvement, but the clinical scales are also prognostic factors to be taken into consideration in the assessment of achieving clinical success. Due to the lower clinical improvement observed in older patients and those with worse clinical and functional scales, it would be preferable to also provide them with the possibility of combined treatments. The ANN predictive model is reasonable and accurate in studying the influence of prognostic factors in achieving clinical success in patients with chronic non-calcific tendinopathy of the supraspinatus treated with ESWT. These parameters analyzed by a predictive model can help the decision-making process for the application of ESWT.

## Figures and Tables

**Table 1:** Demographic and clinical characteristics of group that achieved MCST.

	Group 0 (*n* = 71)	Group 1 (*n* = 138)
**Gender**		
**M**	27 (38.0%)	61 (44.2%)
**F**	44 (62.0%)	77 (55.8%)
**Affected Side**		
**Right**	46 (64.8%)	82 (59.4%)
**Left**	25 (35.2%)	56 (40.6%)
**RM Score T0**		
**1**	15 (21.1%)	32 (23.2%)
**2**	24 (33.8%)	58 (42.0%)
**3**	28 (39.4%)	37 (26.8%)
**4**	4 (5.6%)	9 (6.5%)
**Age**	60.62 (±10.6)	58.9 (±10.1)
**CMS Score T0**	62.2 (±16.6)	64.6 (±15.1)
**VAS T0**	6.1 (±1.9)	6.1 (±1.6)

**Table 2:** Demographic and clinical characteristics. SD: standard deviation.

Assessments	Total 209
Age (mean ± SD), years	59.5 ± 10.2
Gender: Male	88 (42.1%)
Gender: Female	121 (57.9%)
Right Side Affected	128 (61.2%)
Left Side Affected	81 (38.8%)
VAS (mean ± SD)	6.1 ±1.7
Roles and Maudsley Score (mean ± SD)	2.2 ± 0.9
Constant and Murley Score (mean ± SD)	63.8 ± 15.6

**Table 3:** Sample distribution of the ANN model.

	Training Sample	Testing Sample	Exclusion	Total
**Number**	140	67	2	209
**Percentage**	67.6%	32.4%	/	100%

**Table 4:** Mean and standard error of Visual Analog Scale (VAS) scores, Roles and Maudsley (RM) scores, and Constant and Murley scores of patients with chronic non-calcific supraspinatus tendinopathy after treatment with extracorporeal shockwave therapy (ESWT).

	VAS Score	Constant and Murley Score	Roles and Maudsley
**Baseline T0**	6.1 ± 1.7	63.8 ± 15.6	2.2 ± 0.9
**Follow-up T1**	3.1 ± 2.3	79.8 ± 16	1.2 ± 0.7

**Table 5:** Definition of the six predictive factors.

Variable	Definition of Neurons	Type	Value
**Age**	The years of old	Continuous	29–85
**Gender**	Male/Female	Categorical	0, 1
**Affected Side**	Right/Left	Categorical	0, 1
**RM Score T0**	The number of RM score	Categorical	1, 2, 3, 4
**CMS Score T0**	The number of CMS score	Continuous	12–94
**VAS T0**	The number of VAS	Continuous	0–10

**Table 6:** The importance and value of the six independent variables.

No	Variable	Importance	Normalized Importance	*p*-Value	Bivariate Correlations
1	Age	0.281	100%	0.375	−0.62
2	CMS Score	0.224	79.6%	0.002	0.211
3	VAS	0.210	74.8%	0.027	−0.154
4	RM Score	0.149	52.9%	0.037	−0.145
5	Gender	0.104	37.0%	0.001	−0.264
6	Affected Side	0.31	11.1%	0.408	−0.058

**Table 7:** Performance metrics table. Note: AUC, area under the curve; NPV, negative predictive value.

Model Target	Sensitivity (%)	Specificity (%)	AUC (95%CI)	NPV
Vas < 40%	80.7%	72.3%	0.701	96.3%

## Data Availability

The datasets used and data analyzed during the current study will be made available upon reasonable request to the corresponding author (G.S.).

## Abbreviations

AI	Artificial intelligence
ANN	Artificial neural network
CMS	Constant and Murley score
DM	Data mining
ESWT	Extracorporeal shockwave therapy
MLP	Multilayer perceptron
RM	Roles and Maudsley Scale
RC	Rotator cuff
SP	Supraspinatus tendon
SNCCT	Supraspinatus non-calcific tendinopathy
VAS	Visual Analogue Scale
